# Crude extract and fractions from *Eugenia uniflora* Linn leaves showed anti-inflammatory, antioxidant, and antibacterial activities

**DOI:** 10.1186/s12906-018-2144-6

**Published:** 2018-03-09

**Authors:** Tamires Rocha Falcão, Aurigena Antunes de Araújo, Luiz Alberto Lira Soares, Rhayanne Thaís de Moraes Ramos, Isabelle Cristinne Ferraz Bezerra, Magda Rhayanny Assunção Ferreira, Manoel André de Souza Neto, Maria Celeste Nunes Melo, Raimundo Fernandes de Araújo, Andreza Conceição Véras de Aguiar Guerra, Juliana Silva de Medeiros, Gerlane Coelho Bernardo Guerra

**Affiliations:** 1 Department of Biophysics and Pharmacology, UFRN, Av. Senador Salgado Filho, S/N, Campus Universitário, Lagoa Nova, Natal, RN 59072-970 Brazil; 2 Department of Pharmaceutical Sciences, UFPE, Recife, PE Brazil; 3 Department of Pharmacy, UFRN, Natal, RN Brazil; 4 UFRN, Natal, RN Brazil; 5 Department of Morphology, UFRN, Natal, RN Brazil

**Keywords:** *Eugenia uniflora* Linn, Anti-inflammatory, Antinociceptive, Oxidative stress, Antibacterial

## Abstract

**Background:**

This study showed phytochemical composition and evaluates the anti-inflammatory, and analgesic activities of crude extract (CE) and fractions from *E. uniflora* Linn leaves.

**Methods:**

Polyphenols present in crude extract (CE), in aqueous fraction (AqF), and ethyl acetate (EAF) treated fractions from *E. uniflora* Linn leaves were shown by chromatographic analysis in order to conduct a phytochemical characterization. Antibacterial activity was evaluated based on minimum inhibitory concentrations (MICs) determined using the agar dilution method. Doses of 50, 100, and 200 mg/kg of the CE and fractions were applied for conducting in vivo models (male Swiss mice, 8–10 weeks old). The peritonitis experimental model was induced by carrageenan following of Myeloperoxidase activity (MPO), Total glutathione and malondialdehyde (MDA), IL-1β and TNF-α levels by spectroscopic U*V*/VIS analysis. Antinociceptive activity was evaluated based on an abdominal writhing model and hot plate test. The results were statistically evaluated using one-way analysis of variance (ANOVA), followed by Bonferroni’s post-hoc test. The level of statistical significance was *p* < 0.05.

**Results:**

High-performance liquid chromatography with photodiode array detection (HPLC-DAD) detected varying concentrations of gallic acid, ellagic acid, and myricitrin in the CE and fractions obtained from *E. uniflora* Linn leaves (0.05–0.87%*w*/w, 0.20–0.32%w/w, and 1.71–6.56%w/w, respectively). In general, the CE had lower MIC values than the fractions, including the lowest MIC against the MRSA strain. The CE and AqF also significantly reduced leukocyte migration and MPO activity (*p* < 0.05). In addition, AqF significantly reduced IL-1β and TNF-α levels (*p* < 0.05). Furthermore, the CE and fractions exhibited an antioxidant effect (*p* < 0.05) and peripheral analgesic activity (*p* < 0.05).

**Conclusions:**

The CE and fractions from the studied *E. uniflora* Linn leaves exhibited antibacterial, anti-inflammatory, antioxidant, and analgesic activity in the performed assays.

## Background

*Eugenia uniflora* Linn (Myrtaceae) is a species that is native to Brazil and is commonly known as pitanga (Surinam cherry) or pitangueira (Brazilian cherry). It is present as a shrub in the midwest, northeast, southeast, and southern regions of Brazil [1, 2]. In Brazilian folk medicine, the leaves of *E. uniflora* Linn are used in infusions, decoctions, and alcoholic extracts for treating diarrhea, stomachache, colic, intestinal infection, verminosis, fever, flu, cough, bronchitis, anxiety, high blood pressure, and diabetes [3, 4]. Other ethnobotanical studies which did not specify the vegetal part employed have reported the use of this species for treating fever, flu, throat inflammation, dental inflammation, headache, high arterial pressure, and high cholesterol [5].

According to preliminary phytochemical analyses that have been performed, *E. uniflora* Linn leaves contain alkaloids, triterpenes, tannins, flavonoids, and antraquinones [6]. In more detailed studies, hydrolysable tannins (eugiflorins D1 and D2, camptothin A, oenothein B, gemin D, hippomanin A), flavonoids (afzelin, desmanthin-1, myricitrin, quercitrin, and glycosides of myricetin and quercetin), and terpenoids (β-sitosterol, betulinic acid, and centelloside C) have been identified, and isolated in some cases [7].

While many studies have investigated *E. uniflora*, few studies have examined a possible correlation between the phytochemical composition of this dicotyledonous plant and its biological activities. A study by Schumacher et al. (2015) showed that *E. uniflora* Linn leaves reduce the inflammatory infiltrate index in pancreatic islets, maintaining serum insulin levels and hepatic glutathione, and reducing serum lipid peroxidation, as well as reducing the risk for diabetes in non-obese diabetic (NOD) mice [8]. Flavonoid-rich fraction (HE-Bu) obtained from *E. uniflora* leaves decreased TNF-α and IL-1β serum levels, and markedly decreased iNOS and COX-2 protein expression by ileum cells in an in vivo sepsis experimental model [7]. Leaf essential oil and isolated terpenoids from *Eugenia uniflora* Linn presented an analgesic effect in vivo using the mouse abdominal constriction test induced by acetic acid and hot plate test [2].

The aim of the present study was to conduct a phytochemical characterization of *E. uniflora* Linn leaves, while also evaluating the cytotoxicity, antibacterial, anti-inflammatory, and analgesic activity of a crude extract (CE) and prepared fractions from *E. uniflora* Linn leaves.

## Methods

### Herbal material

A sample of *E. uniflora* Linn leaves was collected in the city of Ipojuca (Pernambuco State, Brazil). The species was identified in a herbarium by Dr. Rita de Cássia Pereira and the voucher specimens were deposited at the Agronomy Institute of Pernambuco (IPA), under number 89989. The plant names were verified by http://www.theplantlist.org/.

### Obtaining CE and enriched fractions of *E. uniflora* Linn leaves

***E. uniflora*** **Linn leaves (**50 g) were dried, ground, and then extracted (10%, *w*/*v*) with acetone: water (7:3, *v*/v) by turbo extraction for 20 min at 5 min intervals each with 30 s cycles. The solution was then concentrated under reduced pressure (RV10 Basic, IKA®). The resulting residue was frozen (− 80 °C, 3 d) and then lyophilized (Model L101, Liotop®) to yield CE (with a yield of 10 g). Approximately 10 g of CE were reconstituted in water (100 mL). The resulting aqueous fraction was partitioned twelve more times with 10 mL ethyl acetate. These aqueous (4 g yield) and ethyl acetate (2 g yield) fractions (hereafter referred to as AqF and EAF) were concentrated, frozen, and lyophilized.

### Preparation of solutions for analysis by **high-performance liquid chromatography** with photodiode array detection (HPLC-DAD)

Fifty mg of the CE and fractions were weighed and transferred to 25 mL volumetric flasks. Following the addition of 20 mL of ultrapure water (Elga®) to each flask, the flasks were then transferred to an ultrasonic bath (Ultracleaner, Unique®) to achieve complete dissolution. After 15 min, the CE and fractions were each diluted to 1 mg/mL with ultrapure water. Gallic acid (96% purity, Sigma®) [8], ellagic acid from tree bark (95% purity, Sigma®) [8], and myricitrin (99% purity, Sigma®) [7] were used as reference standards. The CE, fractions, and standards were filtered through a polyvinylidene difluoride (PVDF) 0.45 μm membrane (Macherey-Nagel®) prior to HPLC analysis. The HPLC analyses were performed in triplicate.

### Chromatographic conditions

A Thermo Scientific system (Ultimate 3000 Thermo Fisher Scientific®) equipped with a DAD (Thermo Fisher Scientific®), binary pump (HPG-3x00RS, Thermo Fisher Scientific®), degasser and an autosampler equipped with a 20 μL loop (ACC-3000, Thermo Fisher Scientific®) was used to perform the HPLC analyses. Chromeleon 6.8 software (Dionex®) was used for data acquisition and data processing.

Chromatographic separation was performed with a C_18_ column (250 mm × 4.6 mm inner diameter, 5 μm; Dionex®) that was protected by a guard column made of the same material (Phenomenex®). A gradient elution was achieved by varying the proportion of solvent B (methanol with 0.05%, *v*/v, trifluoracetic acid) to solvent A (water with 0.05%, v/v, trifluoracetic acid) at a flow rate of 0.8 mL/min, according to the following gradient program: 10–25% B (10 min), 25–40% B (5 min), 40–70% B (10 min), 75% B (5 min), and 75–10% B (1 min). Separations were carried out in a column oven at a temperature of 23 ± 2 °C. Wavelengths of 254 nm, 270 nm, and 350 nm were used for detecting ellagic acid, gallic acid, and myricitrin, respectively, according to the maximum absorption measured by DAD.

### In vitro study

#### Antibacterial activity

Antibacterial activity of the CE and fractions was tested against medically important gram-positive and gram-negative bacteria available from the Medical Bacteriology Laboratory, UFRN, Brazil. The gram-positive group included: *Staphylococcus aureus* ATCC 25923, *Staphylococcus epidermidis* INCQS 00016, *Enterococcus faecalis* ATCC 29212, and a methicillin resistant *Staphylocuccus aureus* (MRSA) strain [representative of the Brazilian epidemic clone [9]. The gram-negative group included: *Escherichia coli* ATCC 25922, *Salmonella enteritidis* INCQS 00258, and *Pseudomonas aeruginosa* ATCC. All of these bacteria were previously maintained at − 20 °C and were reactivated on Brain Heart Infusion Broth (BHI, HiMedia®, India) at 37 °C for 24 h. Bacterial suspensions were standardised to a turbidity equivalent to the 0.5 McFarland standard tube before the antibacterial testing was performed.

Minimum inhibitory concentrations (MICs) were determined by the agar dilution method according to the Clinical and Laboratory Standards Institute M07-A9 document [10], with some modifications. First, stock DMSO aqueous solutions (50%, *v*/v) of the CE and fractions (25 mg/mL) were prepared and subsequently filtered through sterile 0.22 μm pore syringe filters (Kasvi®, Brazil). Serial volumes of these stock solutions were then transferred to sterile 15 ml tubes containing Mueller-Hinton agar (MHA, HiMedia®, India) liquefied at 50 °C. The solutions were subsequently homogenized and transferred to sterile Petri dishes (6 mm diameter). The concentration of these samples ranged from 0.039 mg/mL to 2.5 mg/mL, with a final DMSO concentration at the highest sample concentration being (5% v/v). Next, the standardised bacterial suspensions were diluted by 1:10 and 2 μL of each diluted suspension was transferred to media containing the CE or fractions and incubated at 37 °C for 24 h. In addition, a growth control (MHA + innoculum), a solvent control (MHA containing 5% DMSO), and a sterility control (MHA) were incubated at 37 °C for 24 h. Cephalothin, gentamicin and vancomycin (Sigma, St. Louis, MO, USA) were employed as reference antibiotics. The MIC was considered to be the lowest extract or fraction concentration that prevented the visible growth of bacteria.

#### In vivo studies

This study was carried out in strict accordance with the recommendations in the Guide for the Care and Use of Laboratory Animals of the National Institutes of Health. The protocol was approved by the Committee on the Ethics of Animal Experiments of the *UFRN* (*CEUA*, Permit Number: 001/2015).

### Mice

Male Swiss mice, 8–10 weeks old (40 ± 2.0 g) obtained from the UFRN Vivarium Center of Biosciences were maintained under standard conditions (e.g. 12 h light/dark cycle, 22 ± 0.1 °C, and 50–55% humidity) with species appropriately fed and with water provided ad libitum. The animals were acclimated and subjected to a 12 h fasting and water ad libitum prior to the experiments. Euthanasia was performed with a subcutaneous administration of 90 mg/kg sodium thiopental (0.5%, Tiopentax, Cristália, São Paulo, Brazil).

### The carrageenan-induced peritonitis model

Mice were randomly distributed into twelve groups (*n* = 5/group). In order to evaluate the effect of CE and fractions on leukocyte recruitment into the peritoneal cavity, the mice were orally pre-treated with a vehicle (0.9% saline solution)/carrageenan group, CE or Fractions (50, 100 and 200 mg/kg), or Diclofenac (10 mg/kg). After 30 min, 0.25 ml of a 1% carrageenan solution (Sigma-Aldrich, São Paulo, Brazil) was intraperitoneally (i.p.) injected. The sham group received a vehicle (1 mL water/10 g, p.o) and a 0.9% sterile saline solution intraperitoneal injection (0.1 mL/10 g) [11]. Then, the mice were euthanized 4 h later with an overdose of 90 mg/kg sodium thiopental. Three mL of saline solution was then injected into each abdominal cavity and peritoneal fluid was collected and diluted (1:20) in Turk’s solution. A total leukocyte count was performed for each sample with a Neubauer counting chamber. The samples were stored at − 80 °C for subsequent analyses of myeloperoxidase (MPO) activity, as well as malondialdehyde (MDA) and total glutathione levels.

### Determination of myeloperoxidase activity

MPO activity was measured according to the technique described by Krawisz et al. [12]. An aliquot (100 μL) of each sample was diluted in 2 mL of hexadecyltrimethylammonium bromide buffer (HTAB, Sigma Aldrich, São Paulo, Brazil) and homogenized. The samples were sonicated for 5 min before being centrifuged at 10,000 rpm for 15 min at 4 °C, and then subjected to a triple freeze-thaw process. Biochemical measurements were made of duplicate samples. Next, 7 μL of HTAB buffer was added to the blank and supernatant samples in 96-well plates. Then, 200 μl of the staining reagent (o-dianisidine dihydrochloride) was added to each well and absorbance values at 450 nm were recorded by Spectrocopical U*V*/VIS analysis (Biotek, São Paulo, Brazil). MPO enzyme activity was calculated based on interpolation from a standard curve that was generated from MPO from human neutrophils and horseradish peroxidase. A unit of MPO (U) was defined to degrade 1 nmol/min of hydrogen peroxide at 25 °C. Therefore, the results obtained in the assays were expressed as U/μL of sample.

### Determination of total glutathione content

According to the method described by [13], 100 μL of each inflammatory lavage was diluted in a 5% trichloroacetic acid (TCA)/distilled water solution and then homogenized and centrifuged at 10,000 rpm for 15 min at 4 °C. Each standard dilution (20 μL), TCA solution (20 μL, Vetec, São Paulo, Brazil) for the blank, and each sample supernatant (20 μL) were added to 96-well plates in duplicate. In addition, 15 μL PBS-EDTA, 20 μL dithiobisnitrobenzoic acid (DTNB) solution, and 140 μL NADPH were added to each well. After an incubation step at 30 °C for 5 min, 15 μL of an enzyme solution and GSH reductase (Sigma Aldrish, São Paulo, Brazil) were added to each well. Absorbance values at 412 nm were recorded by Spectrocopical U*V*/VIS analysis (Biotek) for 3 min. Total glutathione content was calculated based on interpolations from a standard curve that was generated with purified glutathione (γ-L-Glutamyl-L-cysteinyl-glycine, GSH, Sigma Aldrish, São Paulo Brazil, G4251). The results of these assays are expressed in nmol/μL of sample.

### Determination of MDA content

In order to assess lipid peroxidation, MDA production was measured according to [14]. First, 50 μl of each sample was diluted in 250 μL of 20 mM Tris HCl buffer (Trizma hydrochloride, Sigma Aldrich, São Paulo, Brazil) in distilled water (20 mM, pH 7.4). The peritoneal fluid samples were homogenized and centrifuged at 10,000 rpm for 10 min at 4 °C. Then, 750 μL of a chromogenic reagent (10.3 mM 1-methyl-2-phenylindole in 3:1 acetonitrile) and 225 μL HCl (37%) were added to each sample. After an incubation step in a water bath for 40 min at 45 °C, the samples were centrifuged at 10,000 rpm for 5 min at 4 °C. Absorbance values at 586 nm were recorded with a spectroscopic U*V*/VIS analysis (Biotek, São Paulo, Brazil) and the results were interpolated from a standard curve that was established with 1,1,3,3-tetraethoxypropane (a compound which is hydrolyzed to form MDA when it is incubated with HCl at 45 °C). The results from these assays are expressed as nmol/μl of sample.

### IL-1β and TNF-α assay

Peritoneal fluid (C-carrageenan, D-diclofenac, CE-crude extract, AqF-aqueous fraction, and EAF-ethyl acetate-treated fraction) was stored at − 70 °C after extraction, homogenized and processed as described elsewhere [15]. Levels of IL-1β (detection range: 62.5–4000 pg/mL; lower limit of detection: 12.5 ng/mL recombinant mouse IL-1β), and TNF-α (detection range: 62.5–4000 pg/mL; lower limit of detection: 50 ng/mL recombinant mouse TNF-α) were determined using commercial ELISA kits (R&D Systems, Minneapolis, MN, *USA*), as previously described [16].

First, microtiter plates were coated overnight at 4 °C with antibodies against mouse TNF-α and IL-1β. After the plates were blocked, samples and standards were added at various dilutions in duplicate and incubated at 4 °C for 24 h. The plates were washed 3 times with buffer and antibodies were then added to the wells (biotinylated sheep polyclonal anti-TNF-α, anti-IL-1β, diluted 1:1000 with 1% BSA assay buffer). Plates were incubated at room temperature for 1 h, washed, and 50 μL of avidin-HRP (1:5000) was added. The color reagent o-phenylenediamine (50 μL) was added 15 min later, and the plates were incubated in the dark at 37 °C for 15–20 min. The enzyme reaction was stopped with H_2_SO_4_ and absorbance was measured at 490 nm. Values were expressed in pg/ml.

### Evaluation of antinociceptive activity

#### Hot plate testing

The Hot plate test was used for measuring central analgesic activity and pain was induced by heat [17]. Mice were randomly distributed into eleven groups (*n* = 5/group) and they received: 10 mL/kg saline solution (Normal control group/No treatment), morphine (10 mg/kg, ip), and CE, the AqF or the EAF; with the CE and fractions groups receiving oral doses of 50 mg/kg, 100 mg/kg, and 200 mg/kg of their treatment. Each mouse was then placed on a hot plate (Insight, São Paulo, Brazil) maintained at 55 ± 0.5 °C. The time taken by the animals to jump or lick one of their hind paws was recorded. Latencies were recorded at intervals of 30 min, 60 min, 90 min, and 120 min after administration of the substances.

#### Acetic acid-induced abdominal writhing test

Pain was induced by acetic acid for measuring *peripheral analgesic activity* [18]. Mice were randomly distributed into eleven groups (*n* = 5/group). Two groups were treated with 10 mL/kg oral saline solution (normal control and acetic acid control). One group was treated with oral indomethacin (10 mg/kg). The remaining nine groups were treated with oral doses of CE (50, 100, or 200 mg/kg), AqF (50, 100, or 200 mg/kg), or EAF (50, 100, or 200 mg/kg). Nociception was stimulated with an i.p. injection of acetic acid (0.6% *v*/v) diluted in ultrapure water, 30 min after treating the animals with the CE and fractions, indomethacin and acetic acid control. The normal control received an i.p. injection of saline 10 mL/kg. After the injection of acetic acid or saline, the mice were placed under inverted glass funnels and the number of writhes that were observed over a period of 20 min was recorded.

### Experimental outcomes

The behavior and deaths of animals were recorded during the experiment.

### Statistical analyses

Data from the control group were treated as baseline values. All experimental data were recorded as the mean ± standard error of the mean (SEM). The results were statistically evaluated using one-way analysis of variance (ANOVA), followed by Bonferroni’s post-hoc test. The level of statistical significance was *p* < 0.05. Results analysis and graph generation were performed by using GraphPadPrism version 5.04.

## Results

### Chromatographic analyses of the CE and fractions of *E. uniflora*

**Fig. 1:**
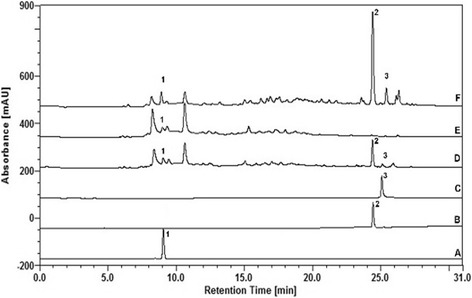
Chromatographic profiles of Gallic acid (A), Myricitrin (B), Ellagic acid (C), CE (D), AqF (E), and EAF (F) of *E. uniflora* Linn leaves as determined by HPLC. CE: crude extract, AqF: aqueous fraction, EAF: ethyl acetate-treated fraction. The retention times (RT) for gallic acid, myricitrin and ellagic acid of these substances were 8.7 min (peak 1), 23.3 min (peak 2), and 25.1 min (peak 3)

Chromatographic analyses were preformed using HPLC. Gallic acid and ellagic acid (monomers of hydrolyzable tannins) and the flavonoid myricitrin were detected in the CE and fractions of *E. uniflora* Linn leaves. The retention times (RT) for gallic acid, myricitrin and ellagic acid in these samples were 8.7 min (Fig. 1a), 23.3 min (Fig. 1b), and 25.1 min (Fig. 1c), respectively. The identities of standards in the samples were confirmed by correlating their RTs. Thus, the peeks corresponding to the standards were determinate in the samples from leaves of *E. uniflora*, as follow: CE (peaks: 1, 2 and 3; Fig. 1d), AqF (peak: 1; Fig. 1e), and EAF (peaks: 1, 2 and 3; Fig. 1f).

Gallic acid, myricitrin and ellagic acid contents in the CE and fractions were also determined in triplicate by using calibration curves of standards (y = 1.3038× + 0.6907; R^2^ = 0.9920; y = 1.4816× – 2.200; R^2^ = 0.9916; y = 3.0007× + 3.9969; R^2^ = 0.9906, respectively for gallic acid, myricitrin and ellagic acid). These values are summarized in Table 1.

**Table 1:** Content of the chemical markers assayed in the CE and fractions of *E. uniflora* leaves as determined by HPLC-DAD

Samples	Gallic acid (%, *w*/w)	Ellagic acid (%, w/w)	Myricitrin (%, w/w)
CE	0.459 (1.99)	0.200 (2.72)	1.713 (0.41)
AqF	0.328 (3.01)	0.035 (3.90)	0.061 (5.15)
EAF	0.872 (0.84)	0.323 (4.05)	6.560 (0.22)

Data presented are the mean (relative standard deviation) of three independent measurements

*CE* crude extract, *AqF* aqueous fraction, *EAF* ethyl acetate-treated fraction

### In vitro activities

#### Antibacterial activity

In the performed antibacterial assays, the CE and fractions of *E. uniflora* Linn inhibited most of the tested bacteria, except MRSA which was not susceptible to the CE at 2.5 μg/mL, or *Escherichia coli* which was not susceptible to any of the tested samples (Table 2). In general, the CE had lower MIC values than the fractions, including the lowest MIC against the *Staphylococcus epidermidis* strain. Normal bacterial growth was observed in all growth (MHA + innoculum) and solvent controls (MHA containing 5% DMSO). No growth was observed in sterility controls (MHA).

**Table 2:** MIC of the CE and fractions of *E. uniflora* Linn against gram-positive and gram-negative bacteria

Bacteria	Samples MIC (μg/mL)^a^					
	CE	EAF	AqF	Cephalothin	Gentamicin	Vancomycin
Gram-positive						
*Staphylococcus aureus* ATCC 25923	1.250 ± 0	2.500 ± 0	2.500 ± 0	0.5 ± 0	ND	ND
*Staphylococcus epidermidis* INCQS 00016	0.313 ± 0	0.938 ± 0.44	0.625 ± 0	0.25 ± 0	ND	ND
*Enterococcus faecalis* ATCC 29212	1.250 ± 0	2.500 ± 0	2.500 ± 0	8.0 ± 0	ND	ND
MRSA	NI	2.500 ± 0	2.500 ± 0	ND	ND	2.0 ± 0
Gram-negative						
*Escherichia coli* ATCC 25922	NI	NI	NI	ND	0.5 ± 0	ND
*Salmonella enteretidis* INCQS 00258	1.250 ± 0	2.500 ± 0	2.500 ± 0	ND	0.125 ± 0	ND
*Pseudomonas aeruginosa* ATCC 27853	1.250 ± 0	1.875 ± 0.88	2.500 ± 0	ND	0.5 ± 0	ND

NI = no inhibition; ND = not determined

^a^Values are the mean seven standard deviation of two replicates

### In vivo activities

All animals presented a perfect state of health and they were included in the experiments. 100% randomised animals of experimental model were included in carrageenan-induced peritonitis model, Hot plate testing and Acetic acid-induced abdominal writhing test. No animals were excluded from the study. No adverse effects were found.

### Leukocyte migration

**Fig. 2:**
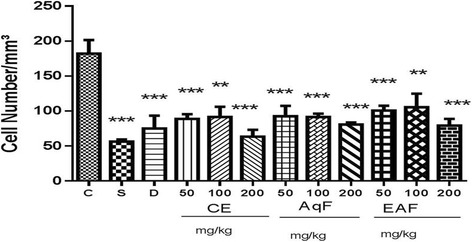
Effects of the CE and fractions of *E. uniflora* Linn at various doses on leukocyte migration in a carrageenan-induced peritonitis model versus the carrageenan group. The results are expressed as the mean ± standard error (*n* = 5). ANOVA test was used to calculate statistical significance. **p < 0.01, ***p < 0.001 versus the carrageenan group (C). S (Sham), C (carrageenan), D (diclofenac), CE (crude extract), AqF (aqueous fraction), EAF (ethyl acetate-treated fraction)

In the leukocyte migration assays, treatment with the CE, AqF and EAF at all doses resulted in a significant inhibition of leukocyte migration versus carrageenan group (*p* < 0.001 or *p* < 0.01, Fig. 2).

### MPO activity

**Fig. 3:**
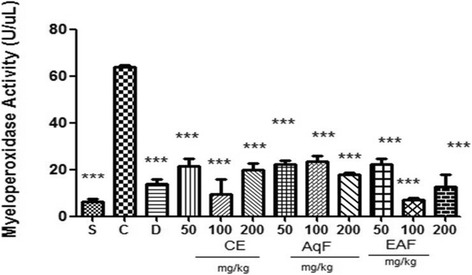
Effect of the CE and fractions (50, 100 or 200 mg/kg) of *E. uniflora* Linn on MPO activity in a carrageenan induced peritonitis model versus the carrageenan group. Data are expressed as the mean ± standard mean error (*n* = 5). An ANOVA test was used to calculate statistical significance, ***p < 0.001 versus the positive control group. S (Sham), C (carrageenan), D (diclofenac), CE (crude extract), AqF (aqueous fraction), EAF (ethyl acetate-treated fraction)

Leukocyte infiltration was assessed with the detection of MPO levels in peritoneal fluid. As shown in Fig. 3, the level of MPO in the positive control group was more than twelve-fold higher (65 U/μL) than the negative control group/saline (5 U/ml) (*P* < 0.001). Following treatment with diclofenac, a significant reduction in MPO levels was observed compared to the positive control group (*P* < 0.001). Significant reductions in the levels of MPO activity at all doses were observed following treatment with the CE, AqF and EAF as a consequence of the number of neutrophils.

### Total glutathione content

**Fig. 4:**
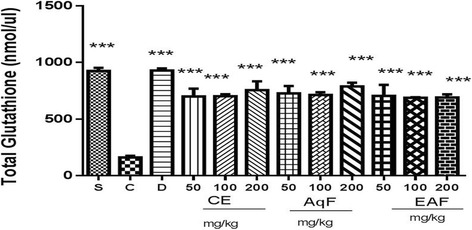
Effects of the CE and fractions (50, 100 or 200 mg/kg) of *E. uniflora* Linn on total glutathione levels (nmol/ul) in a carrageenan-induced peritonitis model versus the carrageenan group. The results are expressed as the mean ± standard mean error (*n* = 5). An ANOVA test was used to calculate statistical significance, ***p < 0.001 versus the positive control group. S (sham), C (carrageenan), D (diclofenac), CE (crude extract), AqF (aqueous fraction), EAF (ethyl acetate-treated fraction)

Total glutathione levels were determined in peritoneal fluid samples in order to evaluate the effect of the CE and fractions of *E. uniflora* Linn on redox homeostasis. In the positive control group, the total glutathione levels decreased approximately 8-fold compared to the negative control group. In contrast, administration of the CE and fractions at doses of 50 mg/kg, 100 mg/kg, and 200 mg/kg all increased the total glutathione levels by 84% relative to the positive control group (*P* < 0.001) (Fig. 4). These results suggest that the CE and fractions of *E. uniflora* Linn mediate antioxidant-related activities.

### MDA content

**Fig. 5:**
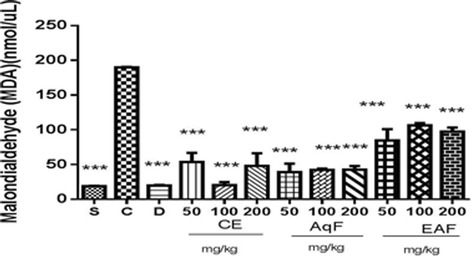
The effects of the CE and fractions (50, 100 or 200 mg/kg) of *E. uniflora* Linn on MDA levels in a carrageenan-induced peritonitis model versus the carrageenan group. S (sham), C (carrageenan), D (diclofenac), CE (crude extract), AqF (aqueous fraction) and EAF (ethyl acetate-treated fraction). Data are expressed as the mean ± standard mean error (*n* = 5). An ANOVA test was used to calculate statistical significance, *p < 0.05), ***p < 0.001 versus the positive control group. ^#^indicates a significant difference between the groups treated with extracts

MDA is one of the main secondary products of lipid peroxidation and is a widely used biomarker to evaluate oxidative stress. MDA is a dialdehyde that is a byproduct of the oxidation of polyunsaturated fatty acids by beta-cleavage of peroxidase and mainly arachidonic acid. As shown in Fig. 5, MDA levels were higher in the positive control group compared to the treated and negative control groups. However, the CE and fractions at all doses were able to significantly reduce MDA levels (*p* < 0.001), thereby indicating that a protective effect on lipid peroxidation is mediated by *E. uniflora* Linn leaves.

### Effect of CE and fractions of *E. uniflora* Linn on cytocines IL-1β and TNF-α

**Fig. 6:**
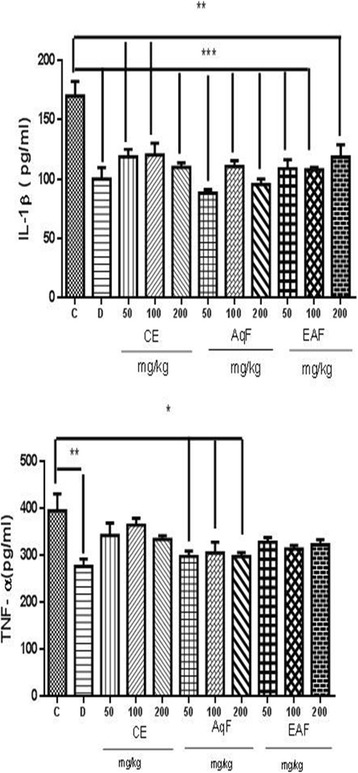
Levels of inflammatory citokynes IL-1β and TNF-**-α** . C (carrageenan), D (diclofenac), CE (crude extract, 50, 100 or 200 mg/kg), AqF (aqueous fraction, 50, 100 or 200 mg/kg), EAF (ethyl acetate-treated fraction, (50, 100 or 200 mg/kg). An ANOVA test was used to calculate statistical significance, *p < 0.05), **p < 0.01 versus the carrageenan control group. ^#^ indicates a significant difference between the groups treated with extracts

The groups subjected to CE and fractions of *E. uniflora* Linn showed decreased levels of proinflammatory cytokine IL-1β (Diclofenac, CE and fractions of *E. uniflora* CE for all doses, *p* < 0.001, except EAF at 200 mg/kg, *p* < 0.01) and TNF- α (Diclofenac, *p* < 0.01, and AgF, all doses, *p* < 0.05) compared to carrageenan control (Fig. 6).

### Evaluation of antinociceptive activity

In the hot plate test, the morphine-treated group exhibited central analgesic action at all time intervals; however, only the dose of the 200 mg/kg AqF-treated group exhibited central analgesic action at the 90 min interval. The AqF-treated group does not have a dose-dependent profile, and therefore this may not be due to pharmaceutical activity of AqF. None of the other groups at the other time intervals exhibited significant central analgesic action (Table 3).

**Table 3:** Evaluation of the analgesic activity of *E. uniflora* CE and fractions at various doses

Administered treatment	Initial pain latency	Pain latency at the indicated time points after administration			
	0 s	30 min	60 min	90 min	120 min
Normal Control/No treatment	13 + 5.0	6.2 + 6.8	4.8 + 3.3	7.8 + 5.1	6.3 + 2.1
Morphine (10 mg/kg)	8 + 2.3	27 + 3.0*	23 + 7.8*	25 + 6.4*	26 + 2.6*
CE					
50 mg/kg	7.6 + 3.2	7.8 + 4.1	11.8 + 7.5	12 + 9.3	8.6 + 6.2
100 mg/kg	8.4 + 2.3	8.4 + 6.0	18.6 + 7.4	6.2 + 6	8.4 + 4.6
200 mg/kg	10.6 + 3.0	8.4 + 5.0	15.6 + 11.1	10.8 + 10.7	12.6 + 6.3
AqF					
50 mg/kg	4.8 + 3.3	13.2 + 5.5	9.6 + 8.1	15.4 + 12.1	14.2 + 9.2
100 mg/kg	5.4 + 2.4	16.2 + 5.6	17.2 + 8.5	9.8 + 5.5	18.4 + 8.2
200 mg/kg	7.6 + 2.7	17.2 + 6.8	12 + 9.7	22.2 + 4.4*	12.6 + 4.6
EAF					
50 mg/kg	7.6 + 2.1	15.2 + 7.7	8.8 + 5.5	6 + 3	10.2 + 6.5
100 mg/kg	5.4 + 2.1	8 + 1.9	5.8 + 2.9	5 + 2.7	3 + 1.5
200 mg/kg	6.2 + 3.5	15.2 + 2.7	8.8 + 2.8	6.2 + 2.9	8.4 + 2.5

**Fig. 7:**
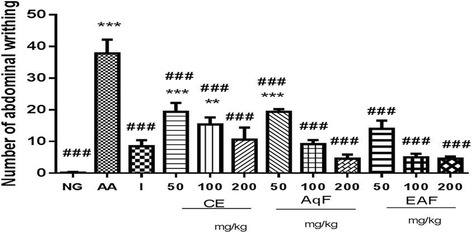
Antinociceptive effects of the Indomethacin, CE and fractions (50, 100 or 200 mg/kg) of E. uniflora Linn at various doses on the number abdominal contortions observed following the administration of acetic acid. Data are expressed as the mean ± standard mean error (*n* = 5). An test was used to calculate statistical significance, *** *p* < 0.001 versus the normal control; ###*p* < 0.001 versus the Acid Acetic. NG (normal control group), AA (acetic acid), I (indomethacin), CE (crude extract), AqF (aqueous fraction), EAF (ethyl acetate-treated fraction)

In the acetic acid induced abdominal writhing assays, administration of the saline in normal control, Indomethacin, CE, AqF and EAF of *E. uniflora* Linn resulted in a significant reduction in the number of abdominal writhes compared to the Acetic Acid control (*p* < 0.001, Fig. 7). Acetic acid showed statistically significant differences, and were also observed for the normal control (*p* < 0.001, Fig. 7).

### Experimental outcomes

No records of behavior change or death were found during the animal experiment models.

## Discussion

The results obtained in the present study establish a scientific basis for the use of *E. uniflora* in folk/traditional medicine. For both in vitro and in vivo tests, the CE and fractions from *E. uniflora* Linn leaves exhibited action on cell viability, antibacterial, anti-inflammatory, and antinociceptive activities.

Myricitrin is the rhamnose glycoside derivative of myricetin, a naturally occurring flavonol that has been extracted from the fruit, bark or leaves of plants. Additionally, myricitrin could also be deglycosylated to the aglycon myricetin [19].

Myricitrin has exhibited several biological functions that represent potential health benefits, including anti-mutagenic [20], antioxidant [21, 22], anti-inflammatory [22–24] and antinociceptive [25] activities in experimental models. Myricitrin has also been found to protect against skin cancer by strongly inhibiting tumour promoting-induced neoplastic cell transformation via restriction of MEK, JAK1, Akt, and MKK4 kinase activity [26]. Furthermore, myricitrin has been shown to attenuate tumour promoting-induced activation of c-fos and activator protein-1 [27], and to inhibit JAK1/STAT3 pathways [28].

In a study by Fiuza et al. (2008), a hydroethanolic extract from *E. uniflora* Linn leaves was subjected to the agar dilution method, and was found to inhibit the growth of *Pseudomonas aeruginosa* strains. However, the associated MIC values 2.18 [29], 4.37 [30], 8.75 [30] and 17.50 mg/ml [29] were approximately 2–7 times higher than the concentrations reported in the present study [31]. In another study which determined MIC values for an *E. uniflora* Linn hydroethanolic extract using the broth microdilution method, the extract was found to inhibit the growth of *P. aeruginosa*, albeit at a lower concentration (10 μg/ml). In addition, the latter extract did not inhibit a *S. aureus* reference strain [32], being in contrast with the present results. Other studies have reported inhibition of *S. aureus* strains, including the reference strain ATCC 25923, with lower MIC values than those reported herein. Inhibition of *E. coli* strains, including *E. coli* ATCC 25922, have also been reported, yet this was not observed in the present study [33, 34]. A similar MIC against *S. aureus* strains, including ATCC 25923, was reported in only one other study, with a MIC of 2.187 mg/ml reported for a hydroethanolic extract with the agar dilution method [35]. These discrepancies may be due to the chemical complexity of the examined samples, especially since the composition of secondary metabolites in vegetal species is dynamic in response to numerous variables related to pre-harvest (harvest site, time, altitude, weather) and post-harvest (preservation method of plant material, extraction method, type of extraction liquid) conditions.

In the performed HPLC analyses, the EAF had higher ellagic acid, gallic acid, and myricitrin content. These results suggest that the EAF had higher phenolic compound content in general, including hydrolysable gallotannins, ellagitanins, and flavonoids. It was previously demonstrated that polyphenols, including gallic acid, ellagic acid, tannins, and flavonoids exhibit antibacterial activity against many bacterial strains [36, 37]. Therefore, we hypothesized that the EAF would be more active due to its higher ellagic acid, gallic acid, and myricitrin content. Despite a previous report that an EAF containing flavonoids and tannins was more active than the extract [35], the CE examined in the present study had a lower MIC value than the other prepared fractions. Thus, it is possible that the antibacterial activity observed for the CE examined herein is due to a synergistic effect of the multiple substances which may have been absent after the fractionation procedure.

Oxidative stress is an event that characterizes many human disorders, including inflammation and cancer. Oxidative stress can result from an imbalance between levels of reactive oxygen species (ROS) and antioxidants that are responsible for cellular defense (e.g. glutathione, superoxide dismutase). In the inflammatory process, oxidative stress is mediated by phagocytes containing MPO, and these result in an overproduction of ROS which overcomes oxidative defense, such as the presence of GSH, a tripeptide normally involved in the prevention of oxidative damage in tissues. Thus, the ability of GSH to maintain a reduced intracellular environment makes it an important antioxidant activity marker.

The beneficial effect of the CE and fractions of *E. uniflora* Linn in the inflammatory process were confirmed based on the observed reduced leukocyte migration. As an important marker of neutrophil migration, MPO activity was also reduced following treatment. The protective effect of the CE on oxidative stress was demonstrated by its ability to prevent the reduction of total glutathione levels and MDA (a marker of lipid peroxidation). The anti-inflammatory capacity of the flavonoid myricitrin was previously demonstrated based on its inhibition of prostaglandin production induced by lipopolysaccharide (LPS) [28]. Myricitrin was also found to inhibit the production of LPS-stimulated nitric oxide, pro-inflammatory cytokines, prostaglandin E2 production, and protein levels of inducible nitric oxide synthase and cyclooxygenase-2 in RAW 264.7 macrophage [23].

In the abdominal writhing model, all extracts at all doses exhibited evident peripheral antinociceptive activity, demonstrating that the decrease in the inflammatory process was accompanied by an evident decrease in pain at the peripheral level. The nociceptive mechanism caused by acetic acid involves different mechanisms such as a release of arachidonic acid metabolites via cyclooxygenase and biosynthesis of prostaglandins and histamines, among others [38]. In the last decade, it has been shown that inflammatory stimuli do not directly stimulate the release of primary hypernociceptive mediators, but that their release is preceded by a cascade of cytokines. There is a cascade release of cytokines that constitutes a link between the injuries and the release of primary hypernociceptive mediators [39]. This concept allows us to understand why the inhibition of one (IL-1β or TNF-α) or several (glucocorticoids) cytokines causes analgesia [39]. Our study was able to show that connection among the CE and fraction, decreased IL-1β and TNF-α levels, and reduced peripheral antinociceptive activity.

On the other hand, the hot plate test consists in evaluating the response of a drug in consequence of a thermogenic stimulus [40]. In this test, the thermal stimulus activates the nociceptors (non-myelinated type C fibers) that transmit the information to specific regions in the Central Nervous System, thus producing a nociceptive response [41]. This test is effective to determine the analgesic activity of opioid agonists [42]. The response of CE and fractions in the hot plate test suggested that it did not demonstrate opioid action.

Polyphenolic constituents such as tannins have antioxidant and anti-inflammatory properties. In a previous study of an aqueous extract of *E. uniflora* Linn, hydrolysable tannins (e.g. gallic acid and ellagic acid) were identified and the extract was applied to an acute diabetic model [8]. The animals that received the extract had a reduced inflammatory infiltrate index, with a decrease in the infiltration of inflammatory cells from 25% to 80% observed in the untreated versus treated animals, respectively. The levels of inflammatory cytokines, especially those of TNF-α, were significantly reduced in the AqF-treated group at all doses, with high ellagic acid and myricitrin content. In macrophages, myricitrin reduced the production of pro-inflammatory mediators such as NO, iNOS, TNF-α, IL-6, and IL-12 through the suppression of NF-κB and STAT1 activation [43]. Ellagic acid pre-treatment decreased the expression of pro-inflammatory cytokines, such as tumour necrosis factor α (TNF-α), interleukin 6 (IL-6), and interleukin 1β (IL-1β). These results suggest that ellagic acid protects against T-cell-mediated hepatitis through TLR and mitogen-activated protein kinase (MAPK)/NF-κB signaling pathways [44].

This anti-inflammatory effect may be partly explained by a reduction in lipid peroxidation, which would imply that an antioxidant effect is mediated by an intracellular reduction in glutathione consumption. The analgesic effects associated with the CE and fractions of *E. uniflora* Linn in the present study are consistent with the antinociceptive activities observed in a study of essential oils that were isolated from *E. uniflora* Linn leaves and inhibited animal constrictions by 48% at an oral dose of 200 mg/kg. The pentane fraction of *E. uniflora* Linn leaves has also been shown to mediate a significant antinociceptive effect which included an inhibition of animal constrictions by 70% at the same dose [2].

Myricitrin was also previously reported to exert a significant analgesic effect in an acetic acid-induced writhing response assay [45]. In the present study, the CE and the fractions induced a significantly greater analgesic effect. This observation may be related to the myricitrin, gallic acid, and ellagic acid contents that were detected in CE and the fraction of *E. uniflora* Linn leaves.

## Conclusions

Regarding the antibacterial effects of *E. uniflora* Linn leaves, a variability of the results reported in the literature in combination with the unexpected findings observed in the present study indicate that further studies are necessary to determine the active antibacterial compound that is present in *E. uniflora* Linn leaves. These studies may involve an evaluation of antibacterial activity in leaf extracts that are collected from different locations, concurrent with a detailed phytochemical analysis. The goal would be to identify which substances are present or absent in active versus less active extracts. The CE and semi-purified fractions of *E. uniflora* Linn leaves exhibited anti-inflammatory and analgesic activities. The treatment doses also inhibited cell migration, as confirmed in MPO assays and showed anti-inflammatory activity with decreased IL-1β levels (CE and all fractions), but AqF (all doses) decreased TNF-α levels. Furthermore, according to the determinations of total glutathione and MDA levels, it is evident that *E. uniflora* Linn leaf extract can mediate antioxidant activity. The AqF may be related to the greater myricitrin, gallic acid, and ellagic acid contents, and induced a significantly greater analgesic effect relative to the CE and the fractions.

## Abbreviations

ANOVA: One-way analysis of variance
AqF: Aqueous Fraction
BAPTA: AM - 1,2-Bis(2-aminophenoxy)ethane-N,N,N′,N′-tetraacetic acid tetrakis(acetoxymethyl ester)
BHI: Brain Heart Infusion Broth
CE: Crude extract
CEUA: Ethics Committee on Animal Use
DTNB: Dithiobisnitrobenzoic acid
EAF: Ethyl Acetate Fraction
GSH: γ-L-Glutamyl-L-cysteinyl-glycine
HPLC-DAD: High-performance liquid chromatography with photodiode array detection
HTAB: Hexadecyltrimethylammonium bromide buffer
i.p.: Intraperitoneal
MDA: Malondialdehyde
MHA: Microbialpolyhydroxyalkanoates
MICs: Minimum inhibitory concentrations
MPO: Myeloperoxidase
MRSA: Methicillin resistant *Staphylocuccus aureus*
NIH: National Institute health
PVDF: Polyvinylidene fluoride
RT: Retention times
SEM: Standard error of the mean
TCA: Trichloroacetic acid
UFRN: Universidade Federal do Rio Grande do Norte
UV: Ultra violete
VIS: Visible
